# Application of a Digital Injury-Surveillance Platform

**DOI:** 10.1001/jamanetworkopen.2025.4799

**Published:** 2025-04-14

**Authors:** Liuyan Zheng, Xiaoyong Li, Zhike Liu, Kun Wang, Qiang Sun, Lei Zhao, Huairong Wang, Jingxian Wu, Huan Yu, Siyan Zhan, Peng Shen, Yiqun Wu

**Affiliations:** 1 Department of Epidemiology and Biostatistics, School of Public Health, Peking University Health Science Center, Beijing, China; 2 Yinzhou District Center for Disease Control and Prevention, Ningbo, China; 3 Key Laboratory of Epidemiology of Major Diseases (Peking University), Ministry of Education, Beijing, China

## Abstract

This cross-sectional study evaluates the validity of a digital injury-surveillance platform in Yinzhou, China.

## Introduction

Injuries pose a considerable burden worldwide, accounting for 9.8% of all-cause disability-adjusted life-years globally.^1^ Reliable surveillance systems play a pivotal role in injury prevention and control. With rapid advances in digital technologies, digital surveillance frameworks have emerged in recent years and been widely applied to infectious diseases^2^ but less commonly in the context of injuries. In Yinzhou, China, a digital platform has been developed that uses machine learning algorithms to process injury information from multiple sources. This study aimed to describe and evaluate the validity of this platform.

## Methods

Informed consent and ethical review were waived for this cross-sectional study because data were deidentified. The study follows the STROBE reporting guideline.

The Yinzhou Digital Injury Surveillance (YDIS) platform was established to systematically collect, analyze, and interpret high-quality injury information. It consists of 3 layers: data sources, information synthesis, and injury control (Figure). Built on the Yinzhou Regional Health Information Platform, which covers more than 98% of Yinzhou residents,^3^ YDIS also incorporates related information from traffic, meteorological data, ambulance dispatch records, fire engine dispatch records, population censuses, and school attendance records. Injury information, including time of occurrence, locations, causes, demographics (eg, age, sex, education level, and occupation), and clinical features (eg, diagnosis, severity, and outcomes), is extracted, integrated, analyzed, and visualized on the platform. Natural language processing techniques are used for retrieving text-based, unstructured information. Unique anonymized identification codes are used to connect various data sources. By leveraging the YDIS platform, collaborative efforts among the government, health care facilities, and the public will be mobilized to implement prevention and control activities across preinjury, injury, and postinjury stages. The YDIS platform has been in operation since 2022 and covers approximately 930 000 residents, with 385 694 injury events reported in 2022 and 2023.

**Figure.**
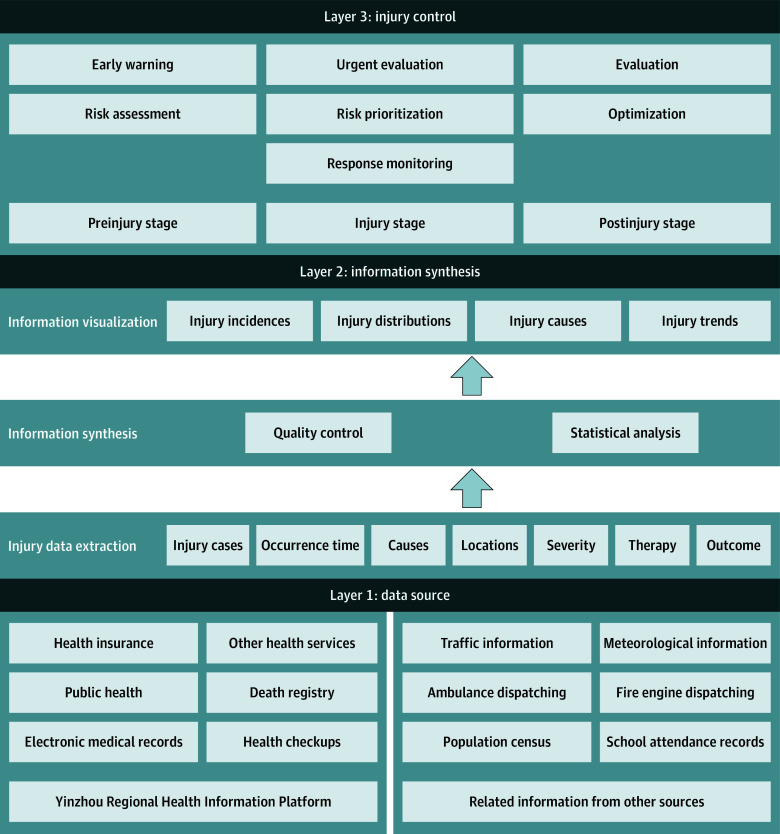
Yinzhou Digital Injury Surveillance Platform Structure

To evaluate the validity of the YDIS platform, we compared it with the traditional manual surveillance system, the National Injury Surveillance System (NISS). Established in 2007, the NISS serves as the criterion standard injury-surveillance platform. Medical records for 14 462 outpatients in the surgery department of NISS sentinel hospitals were selected from 3 randomly chosen months (January, April, and July) in 2022 and then classified as injury or noninjury events by YDIS and NISS separately. Sensitivity, specificity, underreporting rate, misreporting rate, and agreement rate for YDIS were assessed (eMethods in Supplement 1). Analyses were conducted using Python software version 3.10 (Python Software Foundation).

## Results

NISS manual screening classified 6971 injury and 7491 noninjury events, with the YDIS platform accurately identifying 6645 injury and 7301 noninjury events. The YDIS platform had a sensitivity of 95.3%, a specificity of 97.5%, an underreporting rate of 4.7%, and a misreporting rate of 2.5%. The overall agreement between YDIS and NISS was 96.4%.

## Discussion

Using appropriate digital technologies to extract and synthesize information from multiple existing sources is an effective way to acquire reliable data for disease surveillance. Several countries have attempted to adopt digital platforms for injury surveillance.^4^ This cross-sectional study found that the validity of the YDIS platform was favorable and comparable to that of other, previously reported digital injury-surveillance systems.^5^ Additionally, information on the YDIS platform is updated every 2 hours, whereas traditional manual reporting occurs quarterly. This approach saves labor and provides a cost-effective solution.^6^ The implementation of this digital system may represent an advancement in injury prevention, promoting a healthier and safer society through digital means. However, this study has some limitations: the quality of original data, linkage of various sources via individual identifiers, data security, privacy protection, information transparency, equitable data use, and cross-sector collaboration are key elements that require sustained attention and continuous improvement.

## Notes

Supplement 1.
eMethods.

eReferences.

Supplement 2.
Data Sharing Statement
