# Supplementary material for: Application of a Digital Injury-Surveillance Platform
Source: JAMA Netw Open. 2025 Apr 14;8(4):e254799. doi: 10.1001/jamanetworkopen.2025.4799 (PMC11997723; doi:10.1001/jamanetworkopen.2025.4799)
Supplement: Supplement 1. — eMethods. eReferences. [file jamanetwopen-e254799-s001.pdf]

Supplemental Online Content

Zheng L, Li X, Liu Z, et al. Application of a digital injury-surveillance platform. *JAMA Netw Open*. 2025;8(4):e254799. doi:10.1001/jamanetworkopen.2025.4799

eMethods.
eReferences.

This supplemental material has been provided by the authors to give readers additional information about their work.

© 2025 Zheng L et al. *JAMA Network Open*.

eMethods.

Design Observational study.

Setting

The Yinzhou Digital Injury Surveillance (YDIS) platform was developed to systematically collect, analyze, and interpret high-quality injury information in Yinzhou, China.

Data sources

The YDIS platform was built upon the Yinzhou Regional Health Information Platform (YRHIP) and related information from other sources. The YRHIP has been established since 2010, and it has systematically recorded health data for 774,788 residents, more than 98% of the region's population [1]. Nearly all the health-related activities of residents, from birth to death, are covered by the YRHIP system. It has been widely used for estimating disease prevalences [2], identifying risk factors [3], and evaluating intervention effects [4]. In addition, the YDIS platform aggregates data from a variety of sources, such as traffic information, meteorological information, ambulance dispatching, fire engine dispatching, population census, and school attendance records.

Participants

The population covered by YDIS is the same as the YRHIP platform.

To assess the validity of the YDIS platform, the injury events captured by the YDIS were compared with those by the traditional manual surveillance system, the National Injury Surveillance System (NISS). The NISS was established in 2007 and serves as the gold standard in this study. Medical records for all the 14,462 outpatients from the surgical departments of NISS sentinel hospitals were selected from three randomly chosen months (January, April, and July) in 2022, for the comparison.

Measurement

The medical records for the selected 14,462 outpatients were classified as injury and non-injury events by the YDIS and NISS separately. The medical records were scrutinized by specialized staff in the NISS section manually, following standardized procedures. Meanwhile, these records, along with information linked from other resources, were synthesized on the YDIS platform automatically. Natural language processing models were used for integrating text-based unstructured data.

Bias The quality of the original data source determines the accuracy and reliability of the YDIS platform. Accurate and complete data sources ensure that the surveillance platform reflects the true nature of the events, whereas incomplete or erroneous data sources can lead to misleading.

Study size

The YDIS platform covers 774,788 residents. Its validity was assessed using information from 14,462 outpatients.

Statistical methods

The sensitivity, specificity, underreporting rate, misreporting rate, and agreement rate

© 2025 Zheng L et al. *JAMA Network Open.*

of the YDIS platform were calculated using the NISS as the gold standard for comparison. Sensitivity was calculated as the injury cases correctly identified by the YDIS platform divided by all the injury cases. Specificity was calculated as the non-injury cases correctly classified by the platform divided by all the non-injury cases. The underreporting rate was calculated as the number of actual injury cases not captured by the YDIS platform divided by all the injury cases. The misreporting rate was calculated as the number of cases incorrectly classified as injuries by the YDIS platform divided by all the non-injury cases. The agreement rate was calculated as the number of cases correctly classified as injuries by both the YDIS and NISS systems, divided by the total number of injury cases.

© 2025 Zheng L et al. *JAMA Network Open*.

eReferences.

1. Lin H, Tang X, Shen P, et al. Using big data to improve cardiovascular care and outcomes in China: a protocol for the CHinese Electronic health Records Research in Yinzhou (CHERRY) Study. *BMJ Open*. Feb 12 2018;8(2):e019698. doi:10.1136/bmjopen-2017-019698
2. Zeng XY, Liu ZK, Shen P, et al. [Epidemiological study on the incidence of rheumatoid arthritis in adults in Yinzhou district, Ningbo city from 2011-2020]. *Zhonghua Liu Xing Bing Xue Za Zhi*. Aug 10 2022;43(8):1288-1295. doi:10.3760/cma.j.cn112338-20211201-00941
3. Lin HB, Chen Y, Shen P, et al. [Incidence and risk factors of chronic kidney disease in community-based patients with diabetes]. *Beijing Da Xue Xue Bao Yi Xue Ban*. Jun 18 2018;50(3):416-421.
4. Zhao H, Zhuo L, Sun Y, Shen P, Lin H, Zhan S. Thiazolidinedione use and risk of Parkinson's disease in patients with type 2 diabetes mellitus. *NPJ Parkinsons Dis*. Oct 21 2022;8(1):138. doi:10.1038/s41531-022-00406-8

© 2025 Zheng L et al. *JAMA Network Open.*
