# Supplementary material for: Application of a Digital Injury-Surveillance Platform
Source: JAMA Netw Open. 2025 Apr 14;8(4):e254799. doi: 10.1001/jamanetworkopen.2025.4799 (PMC11997723; doi:10.1001/jamanetworkopen.2025.4799)
Supplement: Supplement 2. — Data Sharing Statement [file jamanetwopen-e254799-s002.pdf]

Data Sharing Statement

Zheng. Application of a Digital Injury-Surveillance Platform. *JAMA Netw Open*. Published online April 14, 2025. doi:10.1001/jamanetworkopen.2025.4799

Data
Data available: No

Additional Information
Explanation for why data not available: The data from the Yinzhou Regional Health Information Platform were provided by the Center for Disease Control and Prevention, healthcare institutions, and the Healthcare Security Administration. However, since these agencies have not made these data publicly available, they cannot be disclosed.
